# The impact of the COVID-19 pandemic on the global tuberculosis epidemic

**DOI:** 10.3389/fimmu.2023.1234785

**Published:** 2023-08-29

**Authors:** Dennis Falzon, Matteo Zignol, Mathieu Bastard, Katherine Floyd, Tereza Kasaeva

**Affiliations:** Global Tuberculosis Programme, World Health Organization, Geneva, Switzerland

**Keywords:** tuberculosis, tuberculosis/prevention and control, COVID-19, SARS-CoV-2, epidemiology, pandemics

## Abstract

Tuberculosis (TB) is a major cause of ill health worldwide. Until the coronavirus (COVID-19) pandemic, TB was the leading cause of death from a single infectious agent. COVID-19 has caused enormous health, social and economic upheavals since 2020, impairing access to essential TB services. In marked contrast to the steady global increase in TB detection between 2017 and 2019, TB notifications dropped substantially in 2020 compared with 2019 (-18%), with only a partial recovery in 2021. TB epidemiology worsened during the pandemic: the estimated 10.6 million people who fell ill with TB worldwide in 2021 is an increase of 4.5% from the previous year, reversing many years of slow decline. The annual number of TB deaths worldwide fell steadily between 2005 and 2019, reaching 1.4 million in 2019, but this trend was reversed in 2020 (1.5 million), and by 2021 global TB deaths were back to the level of 2017 (1.6 million). Intensified efforts backed by increased funding are urgently required to reverse the negative impacts of COVID-19 on TB worldwide, made more pressing by ongoing conflicts, a global energy crisis and uncertainties in food security that are likely to worsen the broader determinants of TB.

## Introduction

**Figure 1:**
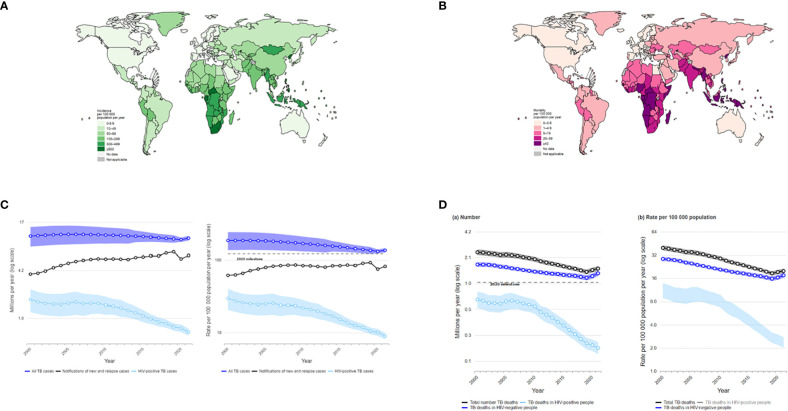
**(A)** Estimated TB incidence rates, 2021. **(B)** Estimated TB mortality rates in HIV-negative people, 2021. **(C)** Global trends in the estimated number of incident TB cases (left) and the incidence rate (right), 2000–2021 (shaded areas represent uncertainty intervals. The horizontal dashed line shows the 2020 milestone of the End TB Strategy). **(D)** Global trends in the estimated number of deaths caused by TB (left) and the mortality rate (right), 2000–2021 (shaded areas represent uncertainty intervals. The horizontal dashed line shows the 2020 milestone of the End TB Strategy).

**Figure 2:**
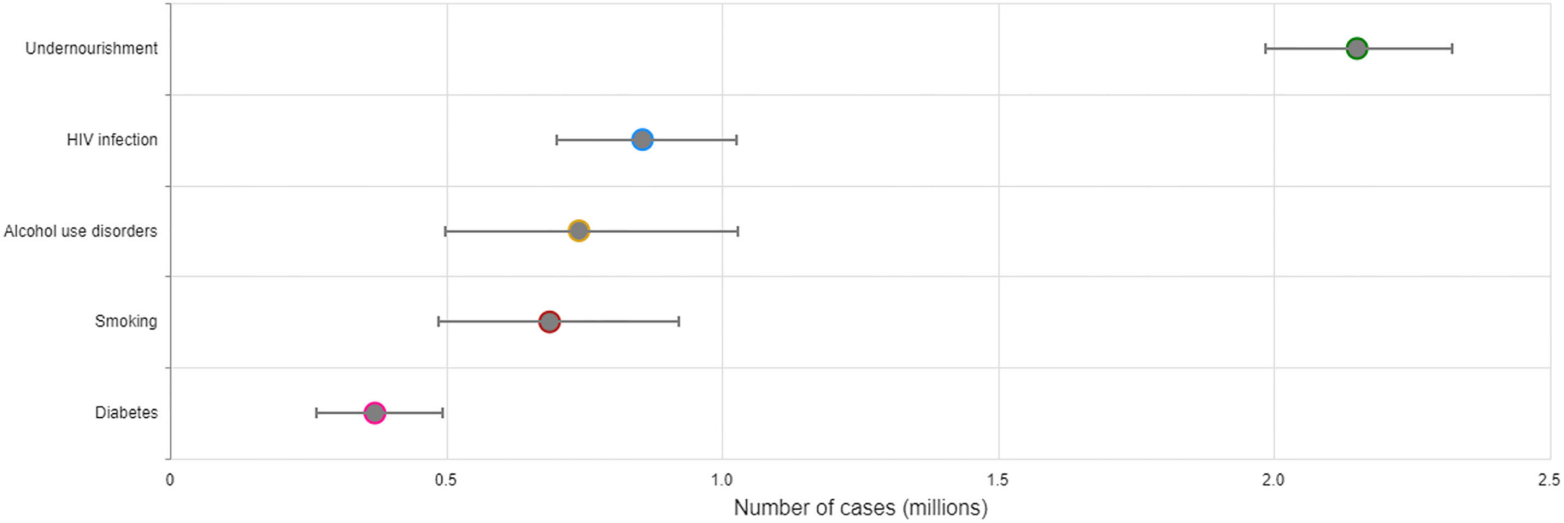
Global estimates of the number of TB cases attributable to selected risk factors, 2021. Sources of data used to produce estimates were: Imtiaz S et al. Eur Resp Jour (2017); Hayashi S et al. Trop Med Int Health (2018); Lönnroth K et al. Lancet (2010); World Bank Sustainable Development Goals Database (http://datatopics.worldbank.org/sdgs/); WHO Global Health Observatory (https://www.who.int/data/gho); and WHO Global TB Programme.

Tuberculosis (TB) is a communicable disease that is a major cause of ill health worldwide (1). It is caused by the bacillus *Mycobacterium tuberculosis*, which is spread when people who have TB disease expel bacteria into the air (e.g. by coughing). About a quarter of the global population is estimated to have been infected with TB bacilli (2), but only about 5-10% of people infected develop disease in their lifetime (3). About 90% of the people who develop TB each year are adults, with more cases among men than women. The disease typically affects the lungs but can affect other sites as well. Without treatment, the death rate from TB disease is high (about 50%). There is a strong geographical bias in the global burden of TB, and much of the TB incidence and mortality is concentrated in Asian and African countries (**Figures 1A, B**). The TB epidemic is strongly influenced by social and economic development and health-related risk factors such as undernourishment, diabetes, HIV infection, alcohol use disorders and smoking (**Figure 2**).

Until the advent of the coronavirus (COVID-19) pandemic, TB was the leading cause of death from a single infectious agent for several years, ranking above HIV/AIDS. COVID-19 has caused enormous health, social and economic impacts since 2020. This includes impacts on the provision of and access to essential TB services, the number of people with TB diagnosed and reported through national disease surveillance systems (TB notification), and the TB epidemiology (TB burden in terms of incidence and mortality).

We present the current situation and recent trends in TB notification and epidemiology worldwide, with a focus on how COVID-19 has impacted upon the main indicators that are used to assess the global TB burden and the response of national health authorities to mitigate it.

## Method

The main indicators used in this article - namely TB notification, TB incidence and TB mortality - were derived primarily from data collected yearly by the World Health Organization (WHO) from national ministries of health as part of its mandate to coordinate international work. Estimates of TB incidence and mortality are based on a well-documented approach (4). For the years 2020 and 2021, TB incidence and mortality were estimated using dynamic models for 28 countries that collectively accounted for 95% of the drop in global TB notifications during these two years. The new methods rely heavily on country and region-specific dynamic models for low and middle-income countries, in the absence of reliable and up-to-date mortality data from national vital registration systems that include standardised coding of causes of death. We compare TB notification and estimates of TB burden with targets set in WHO’s End TB Strategy of 2015 and by the United Nations in 2018 (5, 6). Since 1997, WHO has published annual reports based on the data that it collects from Member States. By 2022, 202 countries and territories with more than 99% of the world’s population and TB cases reported aggregated data to WHO on a series of established indicators. Since the onset of the COVID-19 pandemic, countries can also report monthly or quarterly TB notifications to WHO (7). In addition to TB notification, we also comment on the implementation of TB preventive activities during the pandemic, namely TB preventive treatment and vaccination with bacille Calmette-Guérin (BCG).

## Results

### TB incidence

An estimated global total of 10.6 million people (95% uncertainty interval [UI]: 9.9–11 million) fell ill with TB in 2021, equivalent to a TB notification rate of 134 cases (95% UI: 125–143) per 100 000 population. Similarly, the TB incidence rate (new TB cases per 100 000 population per year) is estimated to have increased by 3.6% between 2020 and 2021, following declines of about 2% per year for most of the past two decades (**Figure 1C**). Among all TB cases in 2021, 6.7% were among people living with HIV. In 2021, eight countries accounted for more than two thirds of global TB cases: India (28%), Indonesia (9.2%), China (7.4%), the Philippines (7.0%), Pakistan (5.8%), Nigeria (4.4%), Bangladesh (3.6%) and Democratic Republic of the Congo (2.9%). The estimated 10.6 million people who fell ill with TB worldwide in 2021 is an increase of 4.5% from the previous year, reversing many years of slow decline. The cumulative fall in the TB incidence rate was 13.5% between 2015 and 2020, but the level of 2021 was only 10% below that of 2015. This was only half-way to the first End TB Strategy milestone of a 20% reduction between 2015 and 2020 and a long way from the second milestone of a 50% reduction by 2025.

### TB mortality

In 2021, there were an estimated 1.4 million TB deaths among HIV-negative people (95% UI: 1.3–1.5 million) and 187 000 (95% UI, 158 000–218 000) among HIV-positive people, for a combined total of 1.6 million. The annual number of TB deaths worldwide fell steadily between 2005 and 2019, reaching 1.4 million in 2019, but this trend was reversed in 2020 (1.5 million), and by 2021 global TB deaths were back to the level of 2017 (**Figure 1D**). Progress previously made towards the first milestone of the End TB Strategy - reducing TB deaths by 35% between 2015 and 2020 - has been reversed and the net reduction from 2015 to 2021 was only 5.9%.

### TB notification

Globally in 2021, 6.4 million people with a new episode of TB (new and relapse cases) were diagnosed and notified. Of these, 83% had pulmonary TB and almost 90% of total notifications were from Asia and Africa. In marked contrast to large increases between 2017 and 2019, there was a substantial fall (-18%) in TB notifications in 2020 compared with 2019, with a partial recovery in TB notifications in 2021 (-10% compared with 2019). However, the gap between the global TB notifications and the estimated incident TB remains similar in 2021 to 2020. Globally, the cumulative total number of people diagnosed with TB and officially reported from 2018 to 2021 is 26 million, only 66% of the 5-year target of 40 million between 2018 and 2022 that was set at the UN high-level meeting on TB in 2018.

### TB preventive activities

The global number of people living with HIV and household contacts of people diagnosed with TB who were provided with TB preventive treatment increased from 1.0 million in 2015 to 3.6 million in 2019, after which there was a sizeable reduction in 2020 (to 3.2 million) followed by an almost complete return to the levels of reporting of 2019 by 2021 (to 3.5 million). Global BCG vaccination coverage decreased from 88% in 2019 to 84% in 2021, reflecting declines in most WHO regions. These trends are likely to be due to concurrent disruptions to health services caused by the COVID-19 pandemic.

## Discussion

The COVID-19 pandemic has caused enormous social and economic impacts, and disrupted healthcare services worldwide. Data reported by countries point to a disproportionate impact on access to essential TB services (8). This has been characterised by pronounced drops in the number of TB cases notified on national information systems. The monitoring of TB notifications at monthly or quarterly intervals allowed a more timely assessment of the impact of the COVID-19 pandemic on TB activities in reporting countries (7).

Decreases in TB notification are likely to reflect two distinct challenges: under-reporting and missed or delayed TB diagnosis on a large scale. Countries have reported disruptions in disease surveillance activities during the pandemic (9, 10). Enhancing health services monitoring and evaluation capacities was one of the most frequently cited needs to be addressed.

Missed and delayed TB diagnosis may have resulted from less opportunities to seek care by people who were unwell during “lockdowns” and prolonged periods of intense activity in primary healthcare clinics. This implies that more people in the community have undiagnosed and untreated TB, and for longer than before, increasing the pool of infectious individuals. Increased transmission and reduced access to proper care could explain at least in part the increments in global and regional TB incidence and mortality observed shortly after notification declined. This is made more plausible by the fact that these epidemiological trends were an abrupt reversal of a steady, albeit slow, decline in the global burden of TB for many years until 2020.

Decreasing TB notification could, however, also indicate less transmission of *Mycobacterium tuberculosis* and less infection. Restrictions in physical mobility and closure of clinics imposed by the authorities of countries during the pandemic may have offset transmission, by as much as 50% according to some modelling studies (11). This would likely happen for short periods of time such as during lockdowns. In such a situation the increased TB mortality could be explained by shortages in timely care of TB patients.

Apart from the effect of pandemic disruptions on TB healthcare services, another concern has been the risk of disease synergy between TB and COVID-19 in the same individual. There is evidence that COVID-19 patients with past and concurrent TB are more likely to have a fatal outcome in high TB burden settings (12, 13). However, it is less clear if TB patients who develop COVID-19 in the course of their illness have a substantially increased risk of dying after adjusting for other risk factors (14). There is also no clear evidence that SARS-CoV-2 infection can increase the progression from TB infection to disease, although no purpose-built studies of the impact of SARS-CoV-2 infection on TB treatment outcomes are known to have been mounted to address this question appropriately.

In addition to the effect on main TB indicators, the COVID-19 pandemic has also impacted negatively on other components of TB programmes in the last three years, such as the provision of TB preventive treatment, vaccination with bacille Calmette-Guérin (BCG) and overall spending on TB (1). Moreover, the negative impact of the disruptions on gainful employment, and key TB determinants such as nutrition and access to care for diabetes and HIV are bound to influence TB incidence and the wellbeing of people affected by TB (**Figure 2**). It is estimated that the COVID-19 pandemic will result in an additional 2.6 million chronically malnourished children by 2022, reversing the decreasing curve for the first time in 3 decades (15). COVID-19 has been associated with both severe COVID-19 at admission and in-hospital mortality in people living with HIV (16). Diabetes control has also been effected in both high and low-resource settings (17, 18).

By mid 2023, close to 770 million confirmed cases of COVID-19 and 7 million deaths had been reported globally since the start of the pandemic (19). In May 2023 WHO declared COVID-19 as an established and ongoing health issue which no longer constitutes a public health emergency of international concern and advised on the transitioning to long-term management of COVID-19 (20). The latest WHO survey on essential health service performance at the end of 2022 registered the first major signs of recovery since the start of the COVID-19 pandemic (10). Recovery from the economic adversities created by the pandemic is likely to take longer in emerging economies and economically disadvantaged groups (21). Intensified efforts backed by increased funding are urgently required to mitigate and reverse the negative impacts of the COVID-19 pandemic on TB. The need for action has become even more pressing in the context of ongoing conflicts, a global energy crisis and associated risks to food security (22), which are likely to worsen some of the broader determinants of TB. The dearth of evidence on disease synergy between COVID-19 and TB is likely to have forfeited opportunities to improve the clinical management of people with both conditions and public health decision making for those at risk. This underlines the need to equip pandemic preparedness plans with research methods for rapid action on major diseases like TB as the world switches gears from emergency phase to contingency strategies for future pandemics.

## Author contributions

All authors listed have made a substantial, direct, and intellectual contribution to the work and approved it for publication.

## Author disclaimer

All authors are current staff members of the World Health Organization (WHO). They alone are responsible for the views expressed in this publication and they do not necessarily represent the decisions or policies of WHO. The designations used and the presentation of the material in this publication do not imply the expression of any opinion whatsoever on the part of WHO concerning the legal status of any country, territory, city or area, or of its authorities, nor concerning the delimitation of its frontiers or boundaries.
